# Cable ties as a method of suicide

**DOI:** 10.29045/14784726.2025.9.10.2.64

**Published:** 2025-09-01

**Authors:** Gary Shaw, Lee Thompson, Graham McClelland

**Affiliations:** North East Ambulance Service NHS Foundation Trust; Northumbria University ORCID iD: https://orcid.org/0000-0001-5279-1412; North East Ambulance Service NHS Foundation Trust ORCID iD: https://orcid.org/0000-0002-0820-1662; Northumbria University ORCID iD: https://orcid.org/0000-0002-4502-5821

Since 2018 the North East Ambulance Service NHS Foundation Trust (NEAS) has collected data on hangings via a comprehensive pre-hospital trauma database. The hanging data includes 54 separate pieces of information, which include patient demographics, patient treatment and detailed information recorded by ambulance staff in the electronic patient care record (ePCR). We have previously published a literature review and service evaluation based on this topic (Shaw et al., 2021a, 2021b).

One of the pieces of information recorded in the audit is the type of ligature used by patients. We have noted a recent worrying trend within the data showing the use of cable ties to cause strangulation. There have been 17 reported cases within the NEAS audit where cable ties have been used as the ligature, with eight (47%) cases of this in 2024. Although the numbers appear low, 30% (5/17) of cases proved fatal, with a further 52% (9/17) requiring immediate medical intervention.

Due to the nature of the ligature and the relative ease of acquiring and using cable ties, we wish to highlight that if patients in distress are found to have mentioned using, or have used, cable ties, there is a high incidence of lethality or serious injury.

There is currently little evidence or literature regarding cable ties being used as a suicide method, with most evidence based upon single case reports (Cecannecchia et al., 2025).

We are unsure if this can be regarded as an anomaly within the data or as an emerging method of self-harm and strangulation.

We urge everybody to be vigilant and aware of the serious nature and implications of patients who have chosen this method.

## Author contributions

GS collected the data to which the letter refers, identified the issue and drafted the letter.

LT collected the data to which the letter refers and reviewed the letter.

GM is supervising GS on his PhD, towards which this work contributes, and reviewed the letter.

## Conflict of interest

GM is on the editorial board of the BPJ.

## Ethics

None required.

## Funding

None.
